# The effect of metformin usage on survival outcomes for hepatocellular carcinoma patients with type 2 diabetes mellitus after curative therapy

**DOI:** 10.3389/fendo.2022.1060768

**Published:** 2022-12-13

**Authors:** Bo Yuan, Jichun Ma, Jing Wang, Jinyong Hao

**Affiliations:** ^1^ Department of General Surgery, Lanzhou University Second Hospital, Lanzhou, China; ^2^ First College of Clinical Medicine, Lanzhou University, Lanzhou, China; ^3^ Cadre Ward Endocrinology Department, Gansu Provincial Hospital, Lanzhou, China; ^4^ Department of Gastroenterology, Lanzhou University Second Hospital, Lanzhou, China

**Keywords:** hepatocellular carcinoma, diabetes mellitus, metformin, overall survival, recurrence-free survival

## Abstract

**Objective:**

Metformin has attracted more attention from researchers for its newly discovered antitumor effects. A meta-analysis was performed to reveal the efficacy of metformin on overall survival (OS) and recurrence-free survival (RFS) for HCC patients with type 2 diabetes mellitus (T2DM) after curative treatment.

**Methods:**

Databases including PubMed, the Cochrane Library, Web of Science, CNKI, Wangfang, and Weipu Database up until 31 May 2022 were searched for relevant studies. STATA 13.0 was used to perform the meta-analysis.

**Results:**

A total of six studies involving 5,936 patients were included in our study. The results from the current study revealed that metformin usage can significantly prolong the 3-year [odds ratio (OR) = 1.50, 95% confidence interval (CI): 1.22–1.83, *p* = 0.000] and 5-year (OR = 1.88, 95% CI: 1.47–2.41, *p* = 0.000) OS and decrease the 1-year (OR = 1.31, 95% CI: 1.08–1.59, *p* = 0.007), 3-year (OR = 1.88, 95% CI: 1.48–2.37, *p* = 0.000), and 5-year (OR = 1.83, 95% CI: 1.40–2.40, *p* = 0.000) recurrence rates.

**Conclusion:**

Metformin treatment significantly prolongs the OS and decreases the recurrence rate for HCC patients with T2DM after curative HCC therapy.

## 1 Background

Metformin, an oral hypoglycemic agent, has become one of the first-line drugs of choice for the treatment of type 2 diabetes mellitus (T2DM) due to its low cost and high efficacy (1). Recently, metformin has attracted more attention from researchers for its newly discovered antitumor effects (2). Growing lines of evidence have shown that metformin can inhibit the progression of cancer, especially in hepatocellular carcinoma (HCC) (3). As we all know, HCC, an aggressive digestive system cancer associated with increasing mortality and morbidity, remains a major global challenge in the field of anticancer treatment (4). However, surgical liver resection, radiofrequency ablation, and liver transplantation have become curative therapy approaches for HCC patients with a 5-year survival rate of 30%–70% (5). There are no effective drugs to prolong their survival outcomes.

T2DM is characterized by metabolic disorder, and the liver plays an important role in glucose metabolism (6). Most HCC patients suffer from a glucose metabolism disorder (7). Recently, more studies have proven that T2DM was associated with a higher risk of HCC and metformin treatment can decrease the risk of HCC for many types of cancers including HCC for patients with T2DM (8). Previous studies have suggested that metformin can prolong overall survival (OS) for cancer patients with DM, which acted as an antitumor drug (9). However, a few studies have shown that metformin increased the OS outcomes of HCC patients with T2DM (10). Therefore, we conducted a meta-analysis with the aim of revealing the efficacy of metformin on OS and recurrence-free survival (RFS) for HCC patients with T2DM after curative treatment.

## 2 Materials and methods

### 2.1 Search strategy

Databases including PubMed, the Cochrane Library, Web of Science, CNKI, Wangfang, and Weipu Database up until 31 May 2022 were searched for relevant studies. The search terms used were as follows: “hepatocellular carcinoma” OR “liver cancer” OR “liver tumor” OR “HCC”, “type 2 diabetes mellitus” OR “diabetes mellitus” OR “T2DM” OR “DM”, “metformin” OR “dimethylguanylguanidine”. Random words and Mesh words were combined in study search. Languages were limited in Chinese and English.

### 2.2 Inclusion and exclusion criteria

Inclusion criteria were as follows: (1) HCC patients with T2DM who were treated with metformin after curative therapy including surgical liver resection and radiofrequency ablation; (2) patients who received metformin for more than 12 months in any dose; (3) all of the randomized controlled trials (RCTs) and case–control studies were included; and (4) survival outcomes including OS and RFS were reported in studies.

Exclusion criteria were as follows: (1) abstracts, review, case reports, editorials, and letters; (2) without sufficient data after contacting the corresponding authors of studies; and (3) duplicate publications.

### 2.3 Data extraction

The data were extracted from eligible studies by two reviewers, including (1) the baseline of included studies, such as author information, the year of publication, country, gender (male/female), metformin usage, mean age, sample size, tumor size (≤5 cm/>5 cm), Child–Pugh grading (A/B/C/D), BCLC (0/A/B/C/D), and type of curative therapy for HCC; and (2) survival outcomes: OS and RFS.

### 2.4 Quality assessment of included studies

The quality of included studies was assessed according to the Newcastle–Ottawa scale (NOS) (11): (1) the selection of cohorts; (2) comparability of cohorts; and (3) the exposure or outcome of the participant. Each item was regarded as 0–4 points. Finally, the total score of each study represented the overall result of quality assessment. Studies with more than seven points were considered as high quality.

### 2.5 Data analysis

STATA 13.0 was used to perform the meta-analysis. Statistic heterogeneity between included studies was assessed by *I* ^2^. If *I* ^2^ was less than 50%, a fixed-effects model was conducted to calculate the combined estimates. Otherwise, the estimates were combined by a random-effects model. Odds ratios (ORs) and its 95% confidence intervals (CIs) were used to compare outcome variants between the metformin and non-metformin groups. Sensitivity analyses were also performed as a possible evaluation of the existing heterogeneity.

## 3 Results

### 3.1 Literature screening

**Figure 1:**
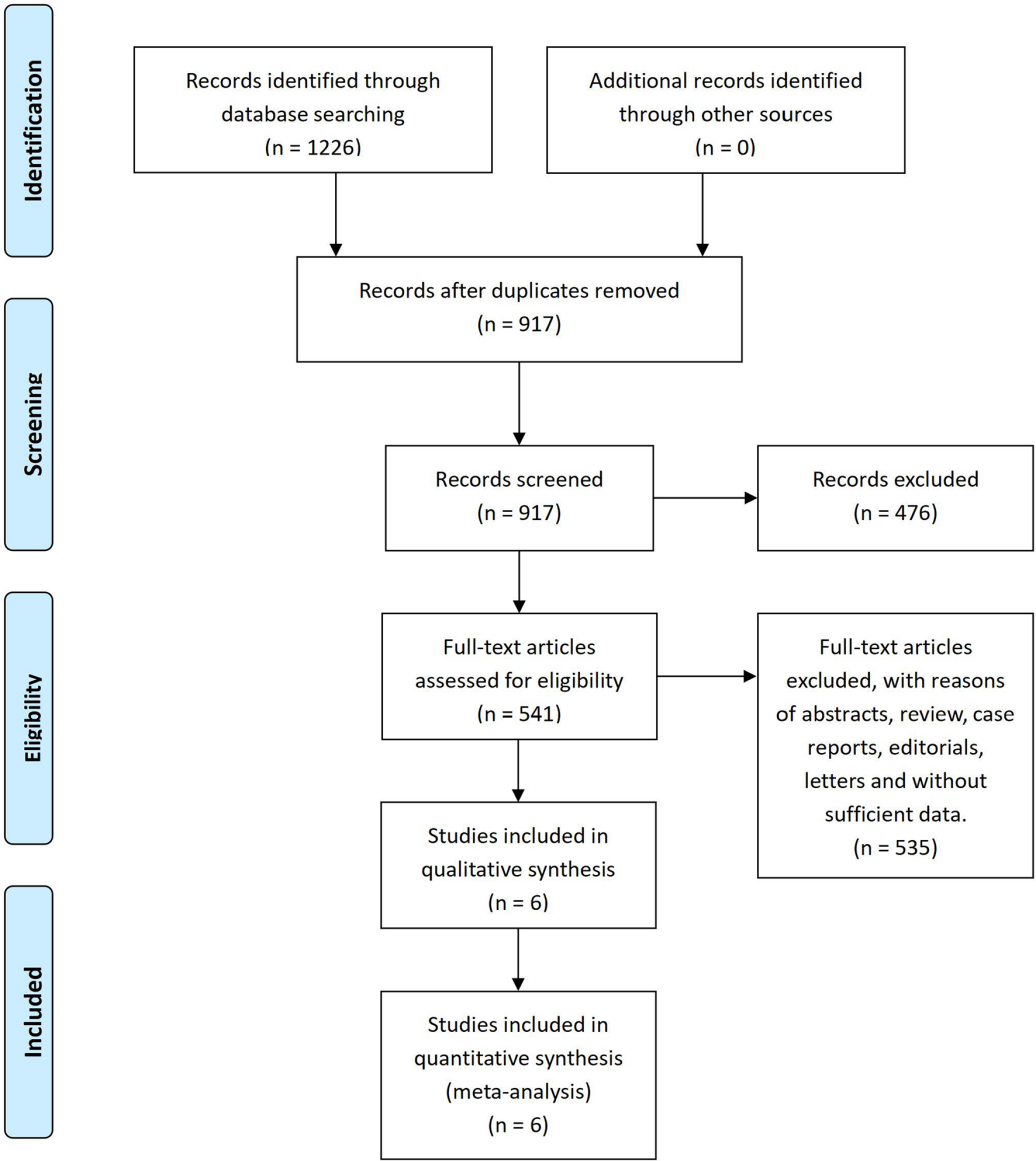
PRISMA flow diagram of the literature search and selection.

After the systematic literature search, 1,226 papers were found from databases by using Mesh and specific search words. A total of 309 duplicates were removed by Endnote X7 and 917 articles were confirmed by screening titles and abstracts. Then, 541 records were assessed in full text for their eligibility. Finally, a total of six studies (12–17) involving 5,936 patients were included in this meta-analysis for qualitative synthesis (**Figure 1**).

### 3.2 The characteristics of the included studies

Among the included studies, three were from China and South Korea. A total of 2,313 HCC patients with T2DM were treated with metformin for more than 12 months and they were regarded as the metformin group. Otherwise, 3,623 patients were treated with other anti-hyperglycemic agents or metformin for less than 12 months, and they were regarded as the non-metformin group. Curative therapy approaches included hepatic resection, radiofrequency ablation, and stereotactic body radiotherapy. The baseline of the included studies is shown in **Table 1**. Quality assessment of the included studies shows that each of the six included studies had a score of more than eight in NOS, which suggests the high methodological quality of each eligibility study.

**Table 1:** The characteristics of the included studies.

Study ID	Country	Gender (male/female)	Metformin usage	Age (mean, years)	Sample size	Tumor size (≤5 cm/>5 cm)	Child–Pugh grading (A/B/C/D)	BCLC (0/A/B/C/D)	Curative therapy for HCC
Chen et al., (14)	China	21/32	Metformin, 750 mg/day	64.2–67.4	21	10/11	10.2 ± 4.5	NR	Radiofrequency ablation
			Sulfonylurea + insulin		32	13/19	9.7 ± 3.4	NR	Radiofrequency ablation
Jang et al., (13)	South Korea	57/19	Metformin, 1,000 mg/day	NR	19	13/6	A: 18, B: 1	A: 12, B: 7	Stereotactic body radiotherapy
			Sulfonylurea + thiazolidinedione + others, dose NR		57	37/20	A: 37, B: 20	A: 36, B: 21	Stereotactic body radiotherapy
Seo et al., (12)	South Korea	598/153	Metformin + sulfonylurea + thiazolidinedione, dose NR	60	533	NR	NR	NR	Hepatic resection
			Sulfonylurea + thiazolidinedione, dose NR		218	NR	NR	NR	Hepatic resection
Chan et al., 2016 (16)	China	3,346/1,264	Metformin, dose NR	64	1,632	NR	NR	NR	Hepatic resection
			Other antidiabetic drugs		2,978	NR	NR	NR	Hepatic resection
Kang et al., (15)	South Korea	214/56	Metformin, dose NR	60.8	45	45/0	A:45	NR	Hepatic resection
			Other antidiabetic drugs		225	225/0	A:225	NR	Hepatic resection
Luo et al., (17)	China	163/13	Metformin + biguanides/sulfonylureas/insulin, dose NR	54.95 ± 8.31	63	NR	NR	A+B: 63	Radical resection
			Biguanides/sulfonylureas/insulin, dose NR	53.77 ± 8.88	113	NR	NR	A+B: 113	Radical resection

NR, not reported; BCLC, Barcelona Clinic Liver Cancer Stage.

### 3.3 Results of meta-analysis

#### 3.3.1 Overall survival

**Figure 2:**
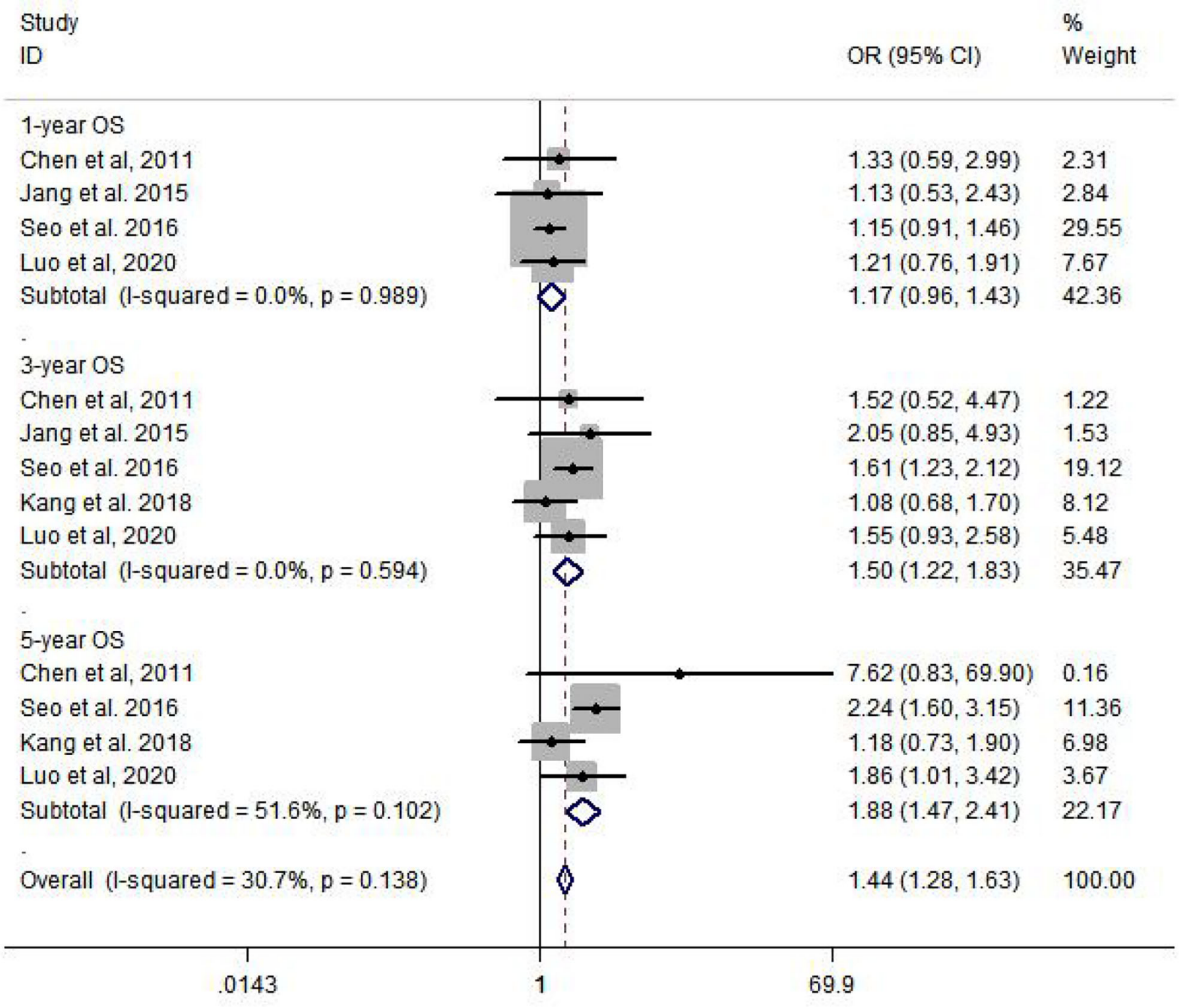
Meta-analysis of overall survival.

Among the six included studies, there is no statistic heterogeneity between studies that reported 1-year OS and 3-year OS. The results of meta-analysis show that there is a significant difference in 3-year OS (OR = 1.50, 95% CI: 1.22–1.83, *p* = 0.000) and 5-year OS (OR = 1.88, 95% CI: 1.47–2.41, *p* = 0.000), compared to HCC patients with T2DM who were not treated with metformin (**Figure 2**).

#### 3.3.2 Recurrence-free survival

**Figure 3:**
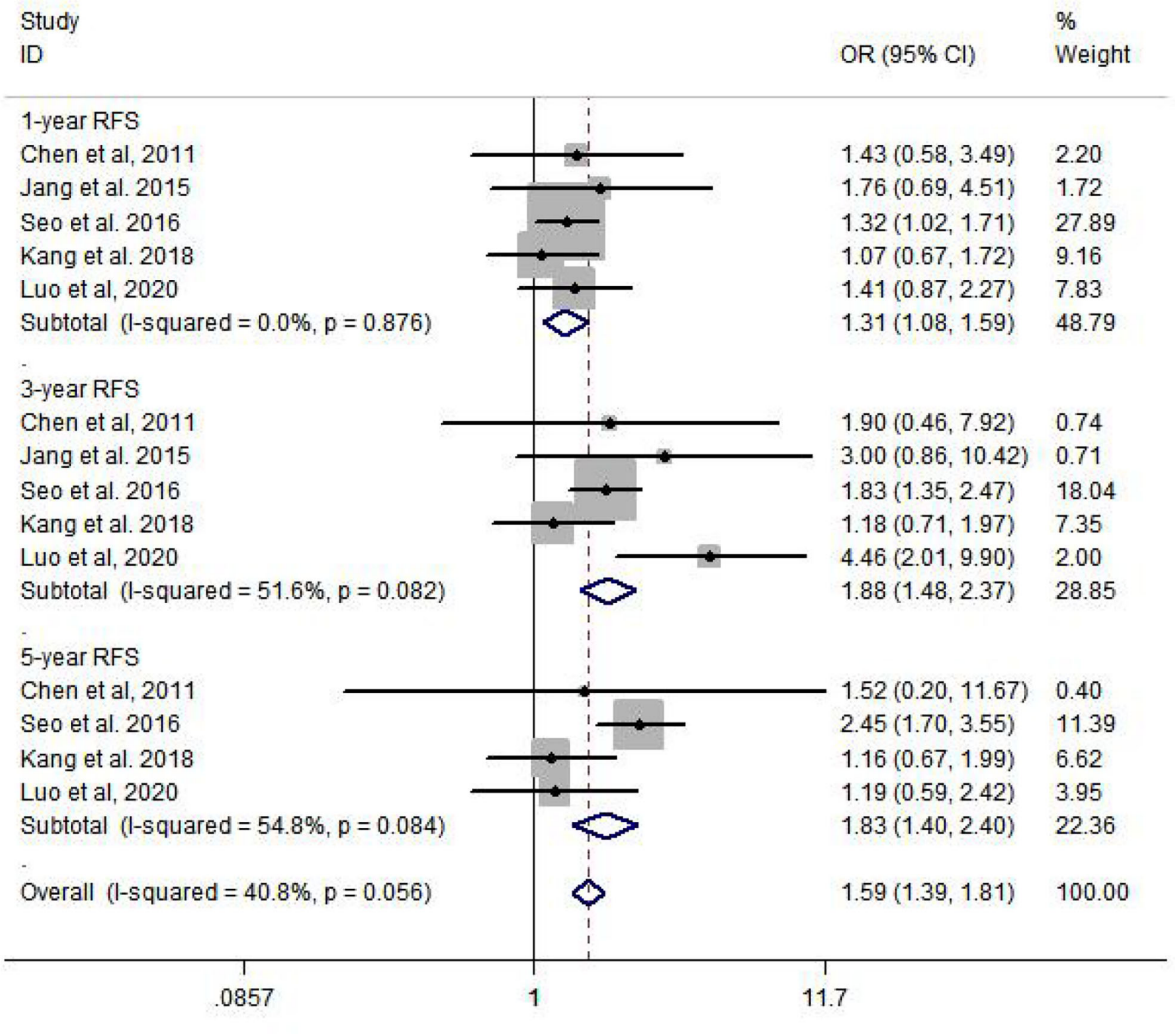
Meta-analysis of RFS.

Compared to the non-metformin group, patients from the metformin group after curative therapy have a significantly longer RFS after 1 year (OR = 1.31, 95% CI: 1.08–1.59, *p* = 0.007), 3 years (OR = 1.88, 95% CI: 1.48–2.37, *p* = 0.000), and 5 years (OR = 1.83, 95% CI: 1.40–2.40, *p* = 0.000) (**Figure 3**).

#### 3.3.3 Subgroup analysis

Subgroup analysis was performed by country and curative therapy approaches in OS and RFS. Compared to HCC patients with T2DM treated without metformin, the metformin group has a higher 3-year OS (OR = 1.49, 95% CI: 1.19–1.86) and 5-year OS (OR = 1.84, 95% CI: 1.40–2.41) among South Korean patients and a higher 5-year OS (OR = 1.86, 95% CI: 1.01–3.42) among Chinese patients. For curative therapy, hepatic resection can prolong the 3-year OS (OR = 1.47, 95% CI: 1.19–1.82) and 5-year OS (OR = 1.84, 95% CI: 1.43–2.36) (**Table 2**).

**Table 2:** Subgroup analysis of OS (OR, 95% CI) .

Subgroup	1-year	3-year	5-year
Country			
China	1.23 (0.83–1.84)	1.54 (0.97–2.45)	1.86 (1.01–3.42)
South Korea	1.15 (0.92–1.44)	1.49 (1.19–1.86)	1.84 (1.40–2.41)
Curative therapy			
RFA	1.33 (0.59–2.99)	1.52 (0.52–4.47)	
SBRT	1.13 (0.53–2.43)	2.05 (0.85–4.93)	
Hepatic resection	1.17 (0.94–1.44)	1.47 (1.19–1.82)	1.84 (1.43–2.36)

RFA, radiofrequency ablation; SBRT, stereotactic body radiotherapy.

Subgroup analysis of RFS showed that there is a significant difference in South Korea in 1-year (OR = 1.28, 95% CI: 1.03–1.60), 3-year (OR = 1.68, 95% CI: 1.30–2.16), and 5-year (OR = 1.98, 95% CI: 1.47–2.66) RFS and hepatic resection in 1-year (OR = 1.28, 95% CI: 1.05–1.58), 3-year (OR = 1.85, 95% CI: 1.45–2.35), and 5-year (OR = 1.84, 95% CI: 1.40–2.41) RFS (**Table 3**).

**Table 3:** Subgroup analysis of RFS (OR, 95% CI).

Subgroup	1-year	3-year	5-year
Country			
China	1.41 (0.93–2.15)	3.77 (1.90–7.48)	1.23 (0.63–2.39)
South Korea	1.28 (1.03–1.60)	1.68 (1.30–2.16)	1.98 (1.47–2.66)
Curative therapy			
RFA	1.43 (0.58–3.49)	1.90 (0.46–7.92)	1.52 (0.20–11.67)
SBRT	1.76 (0.69–4.51)	3.00 (0.86–10.42)	
Hepatic resection	1.28 (1.05–1.58)	1.85 (1.45–2.35)	1.84 (1.40–2.41)

RFA, radiofrequency ablation; SBRT, stereotactic body radiotherapy.

## 4 Discussion

HCC is regarded as the third leading cause of cancer-related deaths worldwide (18). A better survival outcome has been observed only in patients with early-stage HCC who received curative therapy (19). However, most HCC patients are diagnosed in the advanced stages of the disease and undergo non-curative treatment including targeted therapy, transhepatic arterial chemoembolization, and traditional Chinese medicine, which were not satisfactory given the high incidence of progression and recurrence (20). T2DM has been proven to be a risk factor for the development of HCC, which promotes the progression and recurrence of HCC after comprehensive treatment (21). Therefore, whether metformin usage is beneficial to HCC patients with T2DM after curative therapy needs to be further evaluated. Thus, we performed a meta-analysis to provide evidence on the beneficial effects of metformin on the prognosis of HCC patients with T2DM after curative therapy.

In our study, a total of six studies involving 5,936 patients were included to determine the 1-year, 3-year, and 5-year OS and RFS rates. The results from the current study revealed that metformin usage can significantly prolong the 3-year (OR = 1.50, 95% CI: 1.22–1.83, *p* = 0.000) and 5-year (OR = 1.88, 95% CI: 1.47–2.41, *p* = 0.000) OS and decrease the 1-year (OR = 1.31, 95% CI: 1.08–1.59, *p* = 0.007), 3-year (OR = 1.88, 95% CI: 1.48–2.37, *p* = 0.000), and 5-year (OR = 1.83, 95% CI: 1.40–2.40, *p* = 0.000) recurrence rate. Subgroup analysis was performed by country and curative therapy approaches in OS and RFS, which showed that patients who received hepatic resection may have a longer survival time and a lower recurrence rate.

A previous study has revealed that metformin treatment can inhibit HCC cell proliferation and decrease the risk of HCC development in T2DM patients (22). We also found that metformin not only can prolong the OS but also can decrease the recurrence rate for these patients (23). Furthermore, metformin can inhibit energetic metabolism including blood glucose, which may influence the proliferation and migration of HCC cells (24). Moreover, metformin can control the development of T2DM, thereby repairing the liver injury from DM and HCC (25). Sun et al. (26) reported that metformin enhances the antitumor effect by inhibiting growth and invasion and by inducing apoptosis and autophagy in HCC through the PI3K/AKT/mTOR pathway. Another study also showed that metformin activates the Hippo signaling pathway to regulate IL-22-mediated HCC progression (27). All of the above may contribute to increase the OS for HCC patients with T2DM.

Although a comprehensive and systematic literature search was performed, there are several limitations in the present meta-analysis: (1) The original studies included in our study were retrospective studies, which may lead to the heterogeneity between eligibility studies. Large-scale randomized, placebo-controlled trials are necessary to determine the effect of metformin on HCC patients with T2DM after curative treatment. (2) The provided information on the characteristics of the included studies was incomplete; thus, subgroup analysis cannot be performed thoroughly and comprehensively. (3) Most included studies did not report the dose and duration of metformin, which made it more difficult for us to determine the usage of metformin in clinical practice.

In conclusion, metformin treatment significantly prolongs the OS and decreases the recurrence rate for HCC patients with T2DM after curative HCC therapy. Furthermore, large-scale randomized, placebo-controlled trials are needed to provide us with much more high-quality clinical evidence.

## Data availability statement

The original contributions presented in the study are included in the article/supplementary material. Further inquiries can be directed to the corresponding author.

## Author contributions

All authors contributed to the article and approved the submitted version.
